# Exercise for Individuals with Lewy Body Dementia: A Systematic Review

**DOI:** 10.1371/journal.pone.0156520

**Published:** 2016-06-03

**Authors:** Michael Inskip, Yorgi Mavros, Perminder S. Sachdev, Maria A. Fiatarone Singh

**Affiliations:** 1 Exercise, Health and Performance Faculty Research Group, Faculty of Health Sciences, University of Sydney, Lidcombe, New South Wales, 2141, Australia; 2 CHeBA (Centre for Healthy Brain Ageing), School of Psychiatry, University of New South Wales, Randwick, New South Wales, 2031, Australia; 3 Neuropsychiatric Institute, Prince of Wales Hospital, Randwick, New South Wales, 2031, Australia; 4 Sydney Medical School, University of Sydney, Sydney, New South Wales, 2006, Australia; 5 Hebrew SeniorLife, Roslindale, Massachusetts, 02131, United States of America; 6 Jean Mayer USDA Human Nutrition Research Center on Aging at Tufts University, Boston, Massachusetts, 02155, United States of America; University of South Australia, AUSTRALIA

## Abstract

**Background:**

Individuals with Lewy body Dementia (LBD), which encompasses both Parkinson disease dementia (PDD) and Dementia with Lewy Bodies (DLB) experience functional decline through Parkinsonism and sedentariness exacerbated by motor, psychiatric and cognitive symptoms. Exercise may improve functional outcomes in Parkinson’s disease (PD), and Alzheimer’s disease (AD). However, the multi-domain nature of the LBD cluster of symptoms (physical, cognitive, psychiatric, autonomic) results in vulnerable individuals often being excluded from exercise studies evaluating physical function in PD or cognitive function in dementia to avoid confounding results. This review evaluated existing literature reporting the effects of exercise interventions or physical activity (PA) exposure on cluster symptoms in LBD.

**Methods:**

A high-sensitivity search was executed across 19 databases. Full-length articles of any language and quality, published or unpublished, that analysed effects of isolated exercise/physical activity on indicative Dementia with Lewy Bodies or PD-dementia cohorts were evaluated for outcomes inclusive of physical, cognitive, psychiatric, physiological and quality of life measures. The protocol for this review (Reg. #: CRD42015019002) is accessible at http://www.crd.york.ac.uk/PROSPERO/.

**Results:**

111,485 articles were initially retrieved; 288 full articles were reviewed and 89.6% subsequently deemed ineligible due to exclusion of participants with co-existence of dementia and Parkinsonism. Five studies (1 uncontrolled trial, 1 randomized controlled trial and 3 case reports) evaluating 16 participants were included. Interventions were diverse and outcome homogeneity was low. Habitual gait speed outcomes were measured in 13 participants and increased (0.18m/s, 95% CI -0.02, 0.38m/s), exceeding moderate important change (0.14m/s) for PD cohorts. Other outcomes appeared to improve modestly in most participants.

**Discussion:**

Scarce research investigating exercise in LBD exists. This review confirms exercise studies in PD and dementia consistently exclude LBD participants. Results in this cohort must be treated with caution until robustly designed, larger studies are commissioned to explore exercise efficacy, feasibility and clinical relevance.

## 1. Introduction

The worldwide prevalence of dementia is estimated at 44 million people, and is expected to rise to 135 million by 2050 [1]. The majority of dementia cases are attributable to Alzheimer’s disease (AD) estimated at between 50–75% of all prevalence, followed by vascular dementia (20–30%)[2]. However, a significant proportion of dementia cases are conservatively estimated to be attributable to Lewy body Dementia (LBD). The Lewy body dementias encompass both Parkinson’s disease (PD) dementia (3–4%)[3] as well as dementia with Lewy bodies (DLB) (4–5%)[2,4].

The typical symptomology of LBD includes impaired memory, attention and executive function, transient hallucinations and delusions, as well as distinct Parkinsonism, transient losses of consciousness and Rapid eye movement Behaviour Sleep disorder (RBD) [5]. This unique cluster of symptoms means that compared to other more common dementias and idiopathic PD, people with LBD often have a more rapid disease progression, greater rate of hospital admission [6], double the levels of depression [7], and functional decline [8], higher risk of falls [9], higher rates of cognitive fluctuations [10], more visual perception issues [10], lesser quality of life [11], and shorter survival time post-diagnosis [12]. The institutional care of people with LBD has been estimated to be 60% more expensive than for people diagnosed with AD, due predominantly to higher rates of hospitalization [13].

Current treatments for LBD are predominantly pharmacological with a mixture of medications such as acetylcholine-esterase inhibitors (cognitive management), antipsychotics (psychosis management), and dopamine agonists (Parkinsonism management) employed with mixed success [14]. Non-pharmacological treatments are most often occupational interventions to minimize dysfunction in the home environment or physical therapy to improve gait.

Various modalities of exercise have been shown to be beneficial for cognitive and functional outcomes (e.g. gait speed, walking endurance, multi-domain cognition) in dementia populations [15], while also benefiting function (e.g. walking endurance, mobility, and disability) in PD populations [16]. Exercise is often recommended in LBD as well [14], but appears to be included not based on any specific research in this cohort, but rather due to its benefits in these other cohorts. The current gap in evidence stems from an exclusion of LBD populations from both dementia and PD exercise trials, presumably due to their multi-domain cluster of symptoms [17]. Cognitive impairments are a common reason for exclusion from PD trials, while physical impairments are a common reason for exclusion from dementia studies.

Therefore, the aim of this review was to retrieve any studies that explored the effect of exercise or physical activity on individuals with LBD in relation to a variety of outcomes including but not limited to physical, cognitive, psychiatric, physiological and quality of life measures, in order to identify the quantity and quality of the existing evidence base. These results will identify gaps in the literature, which may direct the focus of future robust investigations and clinical practice.

## 2. Methods

The protocol for this review is accessible with registration number (CRD42015019002) at http://www.crd.york.ac.uk/PROSPERO/.

### 2.1 Eligibility criteria

#### Study design

Full-length studies of any design and quality, published or unpublished, and of any language were considered.

#### Population

Human participants of any age with DLB or PDD were eligible, including PD participants with cognitive scores that were indicative of dementia (MMSE<24) but had no reported diagnosis in the study. Animal studies were not included.

#### Intervention

Studies evaluating the isolated effect of exercise (activity prescribed at an effort above activities of daily living to improve wellbeing such as running) or physical activity (low intensity, incidental activity with primary purpose other than improving wellbeing such as cleaning) on any outcome in LBD were included. An intervention could be acute (applied for only one session) or chronic (multiple sessions) but must have been applied separately to outcome testing (i.e. a test of walking endurance could not in itself be an intervention). There were no other restrictions.

#### Comparator and outcome restrictions

None applied in order to minimize risk of excluding studies using atypical terminology for outcomes and comparators.

### 2.2 Database search and strategy

The systematic search was conducted across a wide variety of databases by the primary author (MI), including:

MEDLINE (1946-Current), Premedline, AMED (1985-February, 2015), PSYC info (1806-Current), All EBM review databases [e.g. Cochrane database of systematic reviews (2005-December 2014), ACP journal club (1991–January 2015), Database of Abstracts of reviews of effects (1^st^-quarter 2015), Cochrane central register of controlled trials (January 2015), Cochrane Methodology register (3^rd^-Quarter 2012), Health technology assessment (1^st^-quarter 2015), and NHS economic evaluation (1^st^-quarter 2015)], CINAHL (1981-present), SportsDiscuss (1800-present), Ageline (1966–present), EMBASE (1947-present), Web of Science (MEDLINE entries excluded,1900-current), PEDro (1929–present), ALOIS, Google scholar (100 most relevant samples, 2013-current)

The high-sensitivity search included only ‘Population’ and ‘Intervention’ terms (see S1 Table for strategy) and was simplified when necessary in restricted databases (i.e. Google scholar, PEDro). Email alerts were set up on the major databases after the initial search (28/01/2015) and updated weekly until the last search (28/09/15). The reference lists of relevant review articles were searched for potential articles.

In addition to academic literature databases, a simple stand-alone search engine (Google) and embedded website search engine (Lewy body dementia America (LBDA), Lewy body society UK) were searched weekly throughout the review process to search for lay articles referencing literature.

### 2.3 Study selection

The screening process (conducted by primary author M.I) was sequenced as below:

1. Duplicates were removed through reference management (Endnote X7) software
2. Exclusion by title
3. Exclusion by abstract
4. Full text articles that were deemed ineligible were excluded
5. Full text articles classified as in-doubt or deemed eligible by primary author were reviewed by author (YM) and author (MFS) with subsequent eligible articles included for review.

### 2.4 Data extraction

Data were extracted and analysed for each eligible study by primary author (MI) using pilot tested data forms, adapted for all study designs. A second reviewer (YM) verified the extracted data and subsequent analysis, and any discrepancies in chosen data or analysis were reviewed and resolved prior to tabulation by third reviewer (MFS). Data were extracted at the level of each study (aggregate) and where relevant as individual subject data.

Categories of data were extracted as follows:

1. Study design: Studies were defined as experimental (randomized and nonrandomized control trials (RCT and NRCT), uncontrolled trial (UCT), or case-control) or observational (cross-sectional, prospective or retrospective cohort, case reports, case series) design. Information related to the quality assessment of controlled trials was also collected to complete the Physiotherapy Evidence Database (PEDro) scale criteria. Data collection of observational studies was conducted to enable description as per the Strengthening the Reporting of Observational studies in Epidemiology (STROBE) consensus statement [18] or the Case report (CARE) checklist for case reports [19].
2. Intervention or exposure: exercise modality, volume, frequency, intensity, progression, and duration of program, or in the case of epidemiological data, exposure to physical activity.
3. Cohort: age, sex, diagnosis, years since diagnosis, Mini-mental State Exam (MMSE)/cognitive scores, Unified Parkinson’s Disease Rating Scale (UPDRS)/ mobility scores, co-morbid diseases, medications for PD or dementia, weight/body mass index (BMI), community or non-community dwelling.
4. Outcome: measurement tool or test used, mean and standard deviation (SD) at all time points, effect sizes (ESs), confidence intervals (CIs), mean differences between groups, statistical tests over time and between groups, if available. Where possible, ESs and CIs were calculated from extracted data for each outcome within each study where not provided by authors.

Furthermore, data were collected on the number of PD trials and dementia trials in the abstract and full text stage of the search that were found to be ineligible because they specifically excluded individuals with LBD.

### 2.5 Quality assessment

Quality and risk of bias in intervention trials were assessed with the PEDro scale [20]. Supervision of training interventions was added to the scale to further evaluate quality of the controlled trials, but did not form part of the overall PEDro score. The STROBE checklist for observational studies was selected to assess epidemiological studies [18]. The CARE checklist [19] was selected to assess case reports/case series. Study assessment tools were chosen to be specific to the types of studies retrieved in order to evaluate the quality of each article within the context of each type of study design. Authors MI and YM conducted the quality assessment of included literature, with a plan for any discrepancies in scoring to be resolved through discussion with author MFS in order to reach a consensus. No such discrepancies occurred during this process.

### 2.6 Synthesis of results

A quantitative pooling of data (meta-analysis) was intended at the time of the search if appropriate, but not possible due to a lack of homogenous data and low quality of literature. Sufficient homogenous data was defined as data being available for the minimum number of participants needed to demonstrate similar effects in PD exercise studies with an alpha of 0.05 and a power of 0.8. A qualitative analysis was undertaken for this review. The groupings of data based on the testing domains was analysed for general trends and direction of effect size to be able to make comment on the efficacy of exercise reported in the limited number of participants in context of the bias and quality assessment of the scarce literature found. The authors also considered data presented from similar cohorts (such as Parkinson’s disease, or other dementias) in evaluating the effects of exercise in this cohort.

## 3. Results

### 3.1 Search results

**Fig 1:**
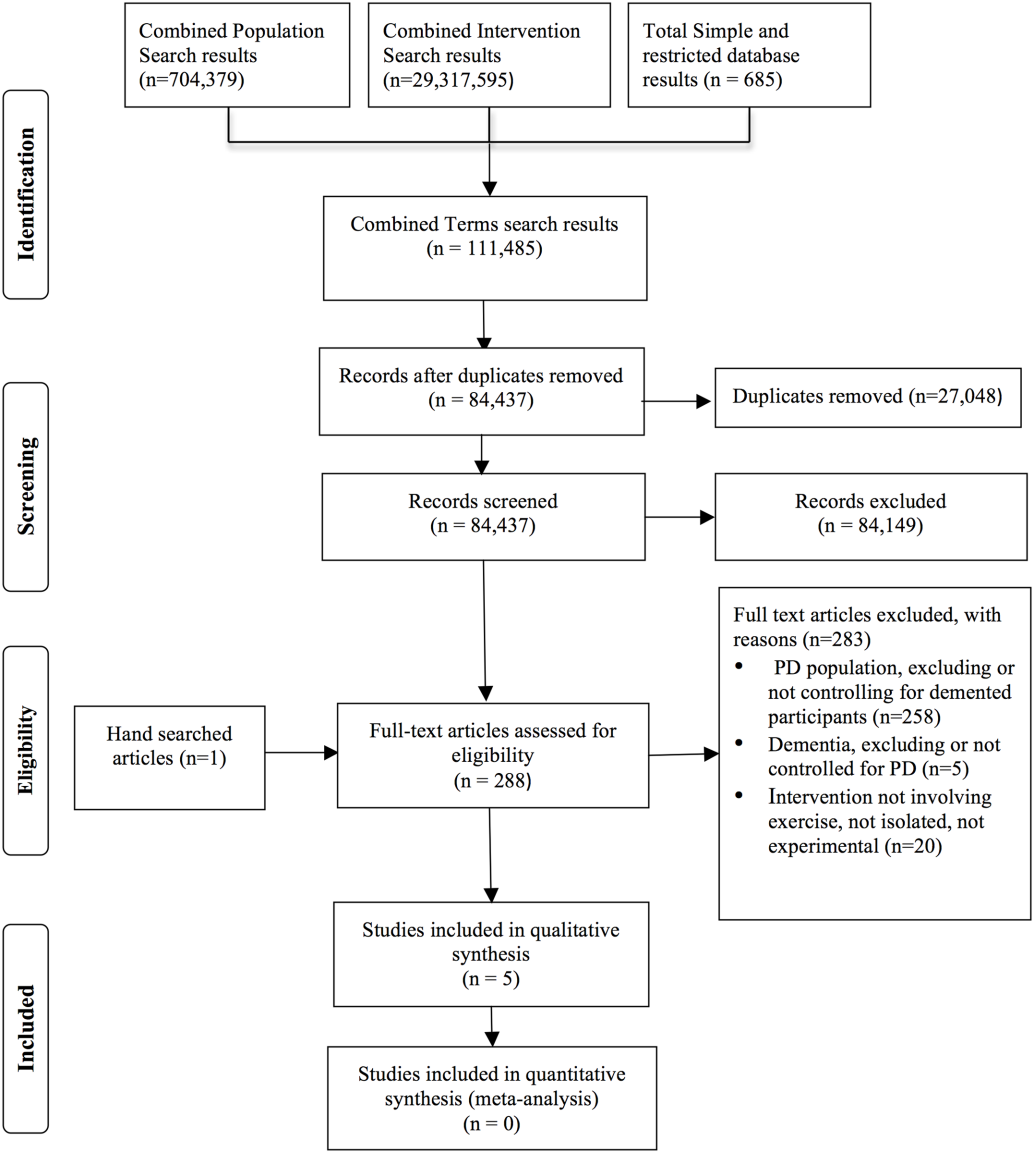
PRISMA flow chart of search.

Fig 1. illustrates the review process in the PRISMA format. The initial search retrieved 111,485 articles that were subjected to the eligibility criteria in the exclusion process. In total, 288 articles were reviewed in full and 283 excluded, with 89.6% of all full texts subsequently deemed ineligible due to exclusion of participants for comorbidities of either dementia or Parkinsonism. Five articles were found eligible including an RCT [21], an UCT [22], and three case reports [23–25], of which two were published. The data analysed in the RCT were a subset (n = 4 participants with PD-dementia) among the 170 participants with dementia in the cohort, accessed with freely available data [21] and assistance from the corresponding author (E.W.T). The last case report [24] was identified in the search process as a poster and after consultation with the corresponding author (C.D) the full, unpublished thesis was attained for analysis [26].

### 3.2 Quality of included literature

The articles were evaluated with either the PEDro criteria [20] (trials) or the CARE criteria [19] (case reports). The RCT by Telenius and colleagues [21] earned a moderate score (5/10) and the UCT by Rochester and colleagues [22] was awarded a poor score (3/10) using the PEDro tool (Table 1). Biases that were common between both of trials included a lack of blinding of the participant and of the therapist. The nature of exercise trials makes it difficult for blinding of therapists due to safety and ethical concerns surrounding professional training regarding interventions, difficulty blinding a visible treatment of a prolonged duration, and duty of care to the participant. This does present the possibility that therapists invested more effort in the active arm and less in the control arm possibly leading to detection bias. The RCT by Telenius and colleagues [21] did not report any attempt to ascertain whether participants knew which intervention was the supposed active arm and which was the control, meaning that in a population where motivation and mood fluctuates frequently participant adherence to the intervention could have been affected. An additional measure added to the quality assessment but not included in overall score PEDro score (out of 10), was supervision of exercise sessions. Health professionals supervised both trials during intervention sessions.

**Table 1:** PEDro score: Experimental studies.

Criteria	Study	
	Telenius et al 2015	Rochester et al. 2009
1. Eligibility criteria were specified	YES	YES
2. Subjects were randomly allocated to groups (in a crossover study, subjects were randomly allocated an order in which treatments were received)	YES	NO
3. Allocation was concealed	YES	NO
4. The groups were similar at baseline regarding the most important prognostic indicators	NO	NO
5. There was blinding of all subjects	NO	NO
6. There was blinding of all therapists who administered the therapy	NO	NO
7. There was blinding of all assessors who measured at least one key outcome	YES	NO
8. Measures of at least one key outcome were obtained from more than 85% of the subjects initially allocated to groups	YES	YES
9. All subjects for whom outcome measures were available received the treatment or control condition as allocated or, where this was not the case, data for at least one key outcome was analysed by “intention to treat”	YES	YES
10. The results of between-group statistical comparisons are reported for at least one key outcome	NO	NO
11. The study provides both point measures and measures of variability for at least one key outcome	NO	NO
**TOTAL PEDRO SCORE**	**5/10**	**3/10**
12. Exercise intervention was supervised	Yes	Yes

The case reports by Dawley [26], Ciro and colleagues [23], and Tabak and colleagues [25] were evaluated with the CARE checklist (Table 2) and all included the majority of essential information outlined in the checklist. Elements often not reported were perspectives from the participants and diagnostic challenges. The only item that all three case reports did not satisfy was the provision of the patient’s history organized as a timeline. This was not considered by the authors to be a crucial component as history was evaluated in a multiple other areas of each report. The quality of the case reports in comparison to CARE criteria was quite high, although the inherent nature of a case report structure means it is highly susceptible to a multitude of biases. These include selection, detection and reporting biases that all arise from the clinical nature of case reports. Achieving set criteria for rehabilitation of a participant within a specific healthcare system rather than a research setting has the potential to cause the therapists to exaggerate treatment outcomes. This can skew the perceived benefit of a therapy when the outcomes of multiple case reports are evaluated together.

**Table 2:** CARE Criteria: Case report studies.

Criteria		Study		
		Ciro et al. 2013	Tabak et al 2013	Dawley 2014
Title	1. The words ‘‘case report’ should appear in the title along with the area of focus	N	Y	Y
Key words	2. 2 to 5 key words that identify areas covered in this case report	Y	Y	N
Abstract	3a. Introduction—what is unique about this case? What does it add to the literature	Y	Y	Y
	3b. The main symptoms of the patient and important clinical findings	N	Y	Y
	3c. The main diagnosis, therapeutic interventions and outcomes	Y	Y	Y
	3d. Conclusion—what are the main ‘take-away’ lessons from this case	Y	Y	Y
Introduction	4. One or two paragraphs summarizing why this case is unique with references	Y	Y	Y
Patient information	5a. De-identified demographic information and other patient specific information	Y	Y	Y
	5b. Main concerns and symptoms of the patients	Y	Y	Y
	5c. Medical, family and psychosocial history including relevant genetic information	Y	Y	Y
	5d. Relevant past interventions and their outcomes	Y	Y	Y
Clinical findings	6. Describe the relevant physical examination and other significant clinical findings	Y	Y	Y
Timeline	7. Important information from the patient’s history organized as a timeline	N	N	N
Diagnostic	8a. Diagnostic methods (such as PE, Laboratory testing, imaging, surveyed	Y	Y	Y
Assessment	8b. Diagnostic challenges (such as access, financial, or cultural)	N	N	Y
	8c. Diagnostic reasoning including other diagnosis considered	N	N	Y
	8d. Prognostic characteristics (such as staging in oncology) where applicable	N	N	N
Therapeutic intervention	9a. Types of intervention (such as pharmacologic, surgical, preventive, self-care)	Y	Y	Y
	9b. Administration of intervention (such as dosage, strength, duration)	Y	Y	Y
	9c. Changes in intervention (with rationale)	Y	Y	Y
Follow-up and outcome	10a. Clinician and patient assessed outcomes (when appropriate)	Y	Y	Y
	10b. Important follow up diagnostic and other results	Y	Y	Y
	10c. Intervention adherence and tolerability (how was this assessed)	Y	Y	Y
	10d. Adverse and unanticipated events	Y	N	Y
Discussion	11a. Discussion of the strengths and limitations in your approach to this case	Y	Y	Y
	11b. Discussion of the relevant medical literature	Y	Y	Y
	11c. The rationale for conclusions (including assessment of possible causes)	Y	Y	Y
	11d. The primary ‘take-away’ lessons of this case report	Y	Y	Y
Patient perspectives	12. When appropriate the patients share their perspective on the treatments they received	N	N	N
Informed consent	13. Did the patient give informed consent? Please provide if requested	Y	Y	Y
	**TOTAL CARE CHECKLIST SCORE (/30)**	**23**	**24**	**26**

### 3.3 Demographic variables

A total of 16 participants (n = 2 controls) were included for analysis (Table 3), consisting of 2/16 with DLB, 10/16 with PD-dementia, and 4/16 with PD with Mild Cognitive Impairment (MCI) that could not be isolated from the dementia cohort in Rochester and colleagues [22]. Mean age was 62 years (57–98 yrs) and the majority (12/16) of the participants were male. Cognitive screening scores were reported as both MMSE [27] scores (n = 14, range 6-26/30) and a Montreal Cognitive Assessment (MoCA) [28] score (n = 1, 17/30). All participants were community dwelling except the participants in Telenius and colleagues [21] (n = 4) who resided in a nursing home. Time since diagnosis of dementia was only reported in the three case reports [23,25,26] and ranged from at the time of screening (0 yrs) up to 2 yrs. Rochester and colleagues [22] reported a mean time since diagnosis of PD only (not dementia diagnosis) as 6 yrs. Most trials reported participant use of neuropsychological medications except the UCT by Rochester and colleagues [22]. There was limited presentation of participant characteristics such as co-morbidities and psychological health, and nutritional status, metabolic health markers, body composition and habitual physical activity levels were searched for explicitly but not reported in any study.

**Table 3:** Cohort Characteristics. Data reported in brackets as mean standard deviation (SD) or as individual values where appropriate. NR—Not reported, PDD—Parkinson’s disease dementia, DLB—dementia with Lewy bodies, MMSE—Mini-mental State Exam score; ranges from 0—30 with higher scores indicative of better cognitive function, UPDRS—unified Parkinson’s disease rating scale (part I—mentation, II—Activities of daily living, III—motor).

Citation	Number of participants	Average Age in years	Sex	Diagnosis	Time since diagnosis in years	MMSE /Cog scores	UPDRS	Hahn’s and Yohr stage	Co-morbid diseases/ conditions	Neuro-psychological medications	Weight/BMI	Residential Status
Rochester et al. 2009	9^A^	75 (6)	M	PDD/PD-MCI	6 (6)^B^	22(3)	Part-III, 44(35.5–47.0)	3 (2.5–3.0)	NR	NR	NR	Community
Ciro et al. 2013	1	73	F	DLB	2	12	NR	4	Low back pain, hip arthritis, osteoporosis, heart palpitations	Rivastigmine, Citalopram, Rasagiline	NR	Community
Tabak et al 2013	1	61	M	PDD	0 ^C^	17 [MoCA^D^]	Part-I 11/16, Part-II 15/52	NR	Deep brain stimulation, total knee replacement	Cardidopa- Levodopa	NR	Community
Dawley, 2014	1	57	M	DLB	1	NR	NR	NR	None	Cardidopa /Levodopa, anti-depressant and anti-hallucinogenic medication	NR	Community
Telenius et al 2015	4	84 (10)	3F, 1M	PDD	NR	16 (7.1)	NR	NR	Average = 2 co-morbid	Average daily medications = 6.5	NR	Nursing home

^A^ 5 participants indicative of dementia, remaining 4 have MCI,

^B^ Time since diagnosis for Parkinson’s disease only,

^C^ participant reported memory concerns 2 years prior, but seemed to have been diagnosed with dementia in the study,

^D^ MoCA = The Montreal Cognitive Assessment was used; score ranges from 0–30 with higher scores indicative of better cognition.

### 3.4 Baseline physical function characteristics

Baseline physical function is presented in Table 4. Habitual gait speed reported for 15/16 participants ranged from 0.36–0.96 m/s (mean = 0.66 m/s, SD = 019 m/s). Maximal gait speed was only reported in 4/16 participants and ranged from 0.49–1.1 m/s. Dual task gait speed (holding a tray) was measured in 9/16 participants with a mean walking speed of 0.65 m/s. Walking distance was seldom recorded, with only one case report recording a six-minute walk distance of 430.86 m.

**Table 4:** Performance based tests of function. N/A = not applicable, NR = not reported.

Study	Measure	EXERCISE		CONTROL				
		Baseline	Outcome	Baseline	Outcome	% change		ES (SD)
**Sit to Stand Function**								
*Single Chair stand*								
Ciro et al. 2013 (n = 1)	*COPM performance (/10)*	1	5			400		
	*COPM satisfaction (/10)*	1	6			500		
	*GAS (-2 to +2)*		0					
*Mutiple Chair stand*								
Dawley 2014 (n = 1)	*30s sit-to-stand (n*. *stands)*	4	8			100		
Telenius et al. 2015 (n = 4) ^A^	*30s sit-to-stand (n*. *stands)*	(#1) 6	(#1) 8	(#3) 7	(#3) 8	N/A	N/A	
		(#2) 5	(#2) 8	(#4) 4	(#4) 4	N/A	N/A	
**Gait speed**								
*Habitual gait speed*								
Rochester et al 2009 (n = 9)	*8m walk (m/s)* ^B^	0.72 ^D^ (0.15)	0.88 ^D^(0.17)			22.2		
Tabak et al 2013 (n = 1)	*10m walk test (m/s)*	0.96	0.92			-4.2		
Dawley 2014 (n = 1)	*7*.*6m walk (m/s)* ^C^	0.8	1.43			78.8		
Telenius et al. 2015 (n = 4) ^A^	*6m walk test (m/s)*	(#1) 0.35	(#1) 0.3	(#3) 0.59	(#3) 0.49	N/A		N/A
		(#2) 0.41	(#2) 0.71	(#4) 0.36	(#4) 0.34	N/A		N/A
*Maximal gait speed*								
Telenius et al. 2015 (n = 4) ^A^	*6m walk test (m/s)*	(#1) 0.49	(#1) 0.5	(#3) 0.93	(#3) 1.11	N/A		N/A
		(#2) 1.1	(#2) 1.54	(#4) 0.71	(#4) 0.57	N/A		N/A
*Dual-talk gait speed*								
Rochester et al 2009 (n = 9)	*8m walk w*. *tray (m/s)*^B^	0.65 ^D^ (0.17)	0.74 ^D^ (0.21)			13.8		
**Walking Endurance**								
*Single task Walk distance*								
Tabak et al 2013 (n = 1)	*2-minute walk (m)*	100.6	129.5			28.7		
Dawley 2014 (n = 1)	*6-minute walk (m)*	480.36	562.05			17.0		
*Dual task walk distance*								
Tabak et al 2013 (n = 1)	*2-minute walk w*. *subtraction task (m)*	60.7	102.7			69.2		
**Balance Function**								
*Scale measures*								
Tabak et al 2013 (n = 1)	*Functional gait assessment (/30)*	13	23					
Dawley 2014 (n = 1)	*MiniBESTest (/28)*	21	25					
Telenius et al. 2015 (n = 4) ^A^	*Berg balance scale (/56)*	(#1) 23	(#1) -27	(#3) 42	(#3)—41	N/A		N/A
				(#4) 29	(#4)—30	N/A		N/A
*Timed Measures*								
Dawley 2014 (n = 1)	*Timed up & go (s)*	15.45	9.05			-41.4		
**Functional Status**								
*Activities of daily living (ADL's)*								
Dawley 2014 (n = 1)	*G-Code*: *mobility* ^AR^ *(% Impaired)*	67	40					
*Instrumented ADL*								
Tabak et al. 2013 (n = 1)	*PDQ-39* ^SR^ *(/156)*	83	70			-15.7		
	*UPDRS-II* ^AR^ *(/52)*	15	6			- 60.0		
Telenius et al. 2015 (n = 4) ^A^	*Barthel index* ^AR^	(#1) 11	(#1) NR	(#3) 11	(#3) 14	N/A		N/A
		(#2) 13	(#2) 12	(#4) 12	(#4) 13	N/A		N/A

^A^ Participant data presented individually (Participant #1–4),

^B^ Unit changed from cm/s to m/s,

^C^ Unit changed from mph to m/s and distance from feet to meters,

^D^ Results rounded to nearest 2 decimal places,

^AR^ -Assessor rated,

^SR^-Subject completed.

COPM—Canadian occupational performance measure. 10-point scale where a higher value indicates better performance/satisfaction, GAS—Goal Attainment scale is scored from -2, -1 (sub-optimal result), 0 (achieved goal), 1, 2 (achieved more than goal), MiniBESTest—Mini Balance evaluation systems test is scored out of 28, with higher scores indicating better function, UPDRS-II—Unified Parkinson’s disease rating scale subscale II (ADL)—is rated out of 52 with lower scores indicating less impairment, PDQ-39 —Parkinson’s disease Questionnaire -39 is rated out of 156 where a lower score indicates less impairment. Barthel Index—a rating scale out of 20, where a higher score indicates more functionality in Instrumented ADL, G—Code—a scale for reporting disability where a higher level of disability is reflected in a higher % range, Functional Gait Assessment—A scale out of 30, where a lower score indicates less function, Berg Balance scale—A scale out of 56 where a higher score indicates more function.

### 3.5 Intervention characteristics

Exercise interventions were varied and included verbal cueing with movement, motor training, stationary cycling, large amplitude bodyweight exercise, high intensity functional exercises and light leisure activities (control group, n = 2). The duration of sessions ranged from 1 to 180 mins, frequency ranged from once only to 5 times/week, and total program intervention ranged from 1 session to 12 wks. The intensity was not reported in three studies, while the cycling intervention reported 50–75% of heart rate maximum, and Telenius and colleagues [21] set a target of performing a maximum of 12 repetitions of given weighted or body weight exercises. Progression method ranged from increasing the complexity of the task to increasing the intensity through velocity or load. Table 5 details the range of exercise interventions employed, with a noticeable absence of interventions related to increasing incidental physical activity in daily life.

**Table 5:** Intervention Characteristics. NR—Not reported.

Citation	Exercise Modality	Frequency (sessions/wk.)	Session/ stimulus duration (minutes)	Program Duration (wk)	Volume (frequency x duration) minutes/wk.	Intensity target	Progression
Rochester et al. 2009	Acute verbal cueing + walking intervals	1 session only	1 ^B^	1 session	~1	NR	No progression
Ciro et al. 2013	STOMP (skill building through task orientated motor practice)	5	120–180	2	600–900	NR	Increasing complexity of task as appropriate
Tabak et al 2013	Stationary cycling	3	40	8	120	50%-75% Heart rate max	5% increase (% heart rate max) in intensity/week
Dawley, 2014	LVST BIG (Lee Silverman voice treatment—Big) Intervention	0.66 ^A^	55	12	36.6 ^A^	NR	Increase in velocity and movement complexity as appropriate
Telenius et al 2015	High intensity functional exercises (exercise group), Light activity (control group)	2	50–60	12	100–120	12RM load	Increase to maintain 12RM intensity

^A^ Average of 8 sessions over 3 months,

^B^ Accumulated estimate of stimulus duration for trials, 12RM = 12 repetition maximum; the maximum amount of weight that can be lifted 12 times only

### 3.7 Outcome measures

The data for the outcome measures discussed below is presented in Table 4. The most commonly reported outcomes were related to physical function measures. These measures were grouped into gait speed, walking endurance, sit-to-stand function, balance and functional status measures. Cognitive, psychiatric, quality of life and physiological measures were rarely reported, and as such grouped into one category.

#### Gait speed

Habitual gait speed changes were reported in 15 participants across four studies (n = 2 participants in a control group). Habitual walking speed of exercise participants (n = 13) increased by 0.18 m/s on average (95% CI -0.02, 0.38m/s).

Maximal walking speed was reported by Telenius and colleagues [21] in four participants (n = 2 in a control group). Group size was insufficient for further statistical analysis.

Rochester and colleagues [22] reported change in dual task (holding a tray) walking speed in 9 PD participants with a mixture of Parkinson’s disease dementia and MCI.

#### Walking endurance

Tests of walking endurance were reported in two case reports involving a total of two participants. Dawley [26] reported a significant post-intervention change in walking distance of 82 m in the sole participant,.

Tabak and colleagues [25] reported considerable improvements in two-minute walking test (2MWT) distance in one participant under single (28.9 m) and dual task conditions (42 m).

#### Sit-to-stand function

Standing function was reported for 6/16 participants across the two case reports [23,26] and the RCT by Telenius and Colleagues (n = 4 participants) [21]. Single chair stand function in Ciro and colleagues (23) improved in one participant using a customized rating scale of performance and satisfaction that was individualized for the participant.

Five participants were tested for multiple (30-second) chair stand ability (n = 2 in control group). Those in the exercise groups (n = 3) performed a mean of 3 (range 2–4) more chair stands in 30-seconds while those in the control group (range 0–1 stands) after training.

#### Balance function

Balance was measured through a range of scale and time measures in 6/16 participants across two case reports and the RCT subset of Telenius and colleagues (21). The timed up & go (TUGT) and MiniBESTest [29] were reported in one participant in the case report by Dawley [26]. The participant improved TUGT by 6.4 seconds and improved 4 points on the MiniBESTest.

The single participant in Tabak and colleagues [25] improved 10/30 points in the Functional gait assessment [30]. 1 of 2 PDD participants allocated to the exercise intervention in Telenius and colleagues [21] improved on the Berg Balance scale [31] by 4 points. The other exercise participant and two control participants changed non-significantly (<1point).

#### Functional status

Basic Activities of Daily Living (ADLs) and Instrumental Activities of Daily Living (IADLs) were reported in 6 participants across two case reports and the RCT by Telenius and colleagues (n = 2 control participants) [21]. Change in the Barthel Index [32] in Telenius and colleagues [21] was inconclusive due to incomplete data. Improvement was reported in the G-code mobility measure [33] employed by Dawley [26], as well as the PDQ-39 [34] and UPDRS-II [35] employed in the case report by Tabak and Colleagues [25]. The UPDRS-II score of the sole participant improved by 9 points.

#### Cognitive, psychiatric, quality of life and physiological outcomes

Outcome data were only collected for neuropsychiatric outcomes in 2/5 studies (5 participants in total, n = 2 control). There was no homogeneity of outcomes for group analysis.

Tabak and colleagues [25] reported improvement in Color Trail Test condition times by 57.5% (condition 1), and 56.7% (condition 2) post intervention for the sole participant. The Parkinson’s Disease Cognitive Rating Scale (PD-CRS) improved 15 points,. UPDRS-I subsection scores for mood and cognition improved considerably 15/16 points.

The RCT subset by Telenius and colleagues [21] reported MMSE scores, Cornell Scale for Depression in Dementia, Quality of Life in Late Stage Dementia (QAULID) and Neuropsychiatric Inventory (NPI) outcomes following intervention. Data for MMSE was incomplete therefore not analyzed, while data for the NPI, Cornell Scale for Depression in Dementia, and QUALID was mixed between groups with incomplete, or non-significant changes.

## 4. Discussion

The aim of this review was to search all available literature reporting the effects of exercise or physical activity on individuals with LBD. Despite an exhaustive search, a total of only 16 participants across five non-robust studies informed the conclusions of this review. Notably, 288 full articles were reviewed and 89.6% subsequently deemed ineligible due to exclusion of participants with co-existence of dementia and Parkinsonism. The dearth of literature increased the difficulty of analysis for the effect of exercise due to small, uncontrolled samples, as well as highlighting the need for higher quality, larger scale research in the LBD population.

The functional capacity of Lewy body dementia participants within the studies is reported to be relatively low in comparison with other similar cohorts. Average habitual gait speed was 0.66 m/s, which was significantly lower than those reported in another LBD cohort of 0.9 m/s [36] and PD cohorts of 1.18 m/s [37]. Furthermore, average dual tasking walking speed was reported in the results to be 0.65 m/s, which was significantly lower than speeds reported in PD populations of 0.97 m/s [38]. The cut off for increased mortality, mobility, disability with activities of daily living, hospitalization and increased dementia risk in geriatric populations is reported as 1.0 m/s [39]. The slower average gait speeds reported in this review fall below this cut off and should be of concern to clinicians as it indicates a trend towards frailty and increased medical complications in this cohort of individuals with LBD.

Despite the low levels of functional capacity, a promising sign in this small and highly varied cohort is the improvements reported in predominantly functional outcomes upon application of an exercise intervention. While the results must be treated with caution due to the low number of participants able to be evaluated and the highly biased study design formats, the review found examples evidence of significant changes in function after exercise. Not all outcomes had comparable data in the literature for expected improvements based on a similar cohort (such as Parkinson’s disease), especially in the cognitive, psychiatric, quality of life and physiological outcomes. There was however some notable improvements in function noted below by this review.

Three studies reported participants improving in gait speed beyond the reported minimally clinical significant change in PD cohorts of 0.06 m/s, and even beyond the moderate clinical significant change of 0.14 m/s [40].

Single case reports demonstrated individually meaningful changes after intervention. The sole participant in the case report of Dawley [26] improved 82m in the six-minute walk test which exceeded minimal and moderate clinically significant changes in geriatric populations of 20-50m [41] and equaled the minimal change that can be reliably detected in PD cohorts [37]. Furthermore, the participant improved on the timed up and go test by 6.4 seconds which exceeded the minimal change reliably detectable in PD cohorts (3.5–4.8 seconds [42,43]). The participant in the case report by Tabak and colleagues [25] significantly exceeded the large important clinical change for the daily activities subsection of the UPDRS (section-II) of 4.3–4.6 points [44] by improving by 9 points following exercise.

No previous reviews of exercise in LBD exist to our knowledge. Other literature supports the preliminary findings in LBD above, and has established the effectiveness of exercise in populations that have similar symptoms to LBD, such as PD and non-motor dementias. For example, exercise has an ES ranging from 0.5 to 2 [15] for outcomes including cognition, function, fitness and strength in dementia cohorts, while PD studies report a mean ES of 0.47 for functional outcomes [16]. Logically, it is reasonable to theorize that exercise may have similar effects on LBD populations who report both dementia and Parkinsonian symptoms, but the existing literature is insufficient to establish ESs with confidence. Conversely, it is possible that the complexity of co-existing cognitive and motor impairments would make exercise too difficult to implement or minimize its efficacy in LBD. Until robust RCTs are conducted in sufficiently powered trials, no firm conclusions can be drawn in this regard.

### 4.1 Strengths of this review

This is the first review of exercise in LBD. It was rigorously executed across 19 reputable databases and was continually updated over a period of eight months through RSS and email search updates. All types of full-length articles in any language were reviewed regardless of publication status. Authors of included and excluded papers were contacted for additional data or information if warranted, which ensured all available information on LBD cohorts could be included in the review.

The review protocol was registered prospectively with PROSPERO (CRD42015019002) and conformed to PRISMA guidelines in all applicable areas [45].

### 4.2 Limitations of this review

A limitation of the methodology in this review was the use of only the primary author to conduct the searches up to the final stage of full article review and analysis.

The scarce and low-quality literature available for analysis has been identified as a limiting factor for any reported conclusions in this review despite the comprehensive search strategy conducted by the authors.

### 4.3 Conclusion

The effect of exercise in individuals with LBD was evaluated in this review. The limited data available in small, uncontrolled studies suggest further research in larger cohorts needs to be conducted to evaluate any benefits reported in this small amalgamated group of individuals with LBD before any judgments about the efficacy of exercise can be made. The effect of exercise on other outcomes measured in this review including cognition, psychiatric, quality of life and physiological outcomes remain unclear. A crucial finding of this review is that the overwhelming majority of literature in related populations excludes LBD individuals from research studies due to a cluster of symptoms that is multi-domain and seen to be confounding to research data.

Exercise prescription is an intervention that requires not only sustained physical exertion but also cognitive engagement. The implementation of exercise in a LBD cohort may be a task that is complicated by a cognitive impairment coupled with physical symptoms of Parkinsonism and autonomic concerns such as orthostatic hypotension. An intervention in this cohort needs to be tactful in the delivery of exercise, as the benefits demonstrated with various exercise intervention in PD cohorts may not be transferable to LBD cohorts if cognition is limiting the drive, engagement or comprehension of participants. Similarly, benefits of various exercise interventions in other dementias such as AD may not be feasible in a LBD cohort with Parkinsonism limiting speed, amplitude, length and complexity of movement. An exercise intervention similar to those seen in PD cohorts used to target the physicality of LBD, but delivered in a dementia friendly format is likely to be the most viable modality to investigate in future research.

This review highlights the importance and need for conducting controlled trials that are preferably randomized, in larger sample sizes of LBD cohorts to reliably evaluate the efficacy, feasibility and relevance of exercise in this vulnerable population.

## Supporting Information

S1 Table Search strategy for systematic review. Search terms where simplified where necessary for certain search engines.
(PDF)

S1 Fig PRISMA Checklist. (PDF)
