# Supplementary material for: Exercise for Individuals with Lewy Body Dementia: A Systematic Review
Source: PLoS One. 2016 Jun 3;11(6):e0156520. doi: 10.1371/journal.pone.0156520 (PMC4892610; doi:10.1371/journal.pone.0156520)
Supplement: S1 Table — Search terms where simplified where necessary for certain search engines. (PDF) [file pone.0156520.s001.pdf]

S1 Table - Search strategy for systematic review

Intervention	Population
Exercise OR 'leisure activity' OR 'leisure activities' OR 'recreational activities' OR 'recreational activity' OR 'occupational activity' OR 'occupational activities' OR bicycle OR 'balance training' OR 'strength training' OR 'aqua aerobics' OR 'water aerobics' OR boxing OR gym OR dance OR dancing OR 'weight training' OR weightlifting OR swim* OR 'weight lifting' OR 'cardiovascular training' OR physiotherapy OR circuit training OR flexibility OR stretching OR 'power training' OR physical therapy OR hydrotherapy OR heartmoves OR 'heart moves' OR cycling OR 'physical activity' OR 'group exercise' OR 'exercise group' OR aerobic* OR 'resistance training' OR training OR walk* OR 'Incidental activity' OR 'incidental activities' OR 'resistance activities' OR 'resistance activity' OR rowing OR skipping OR prt OR 'Progressive resistance training' OR 'leisure time activities' OR 'leisure time activity' OR 'exercise class' OR 'lawn bowls' OR running OR jogging OR sport OR 'cardiovascular exercise' OR 'endurance training' OR 'endurance exercise' OR 'isometric training' OR 'isometric exercise' OR pilates OR yoga OR 'Qi gong' OR 'chi gong' OR 'tai chi' OR 'martial arts' OR calisthenics OR calisthenics OR activit*	'Lewy Body' OR 'Lewy Bodies' OR 'Lewy disease' OR Parkinson*

Coding of strategy was changed according to each database
